# Chemical Risks, Genotoxicity, and Oxidative Stress in Healthcare Workers

**DOI:** 10.3390/toxics13030189

**Published:** 2025-03-06

**Authors:** Ayşe Coşkun Beyan, Esra Emerce, Gamze Tuna, Gül Hüray İşlekel

**Affiliations:** 1 Faculty of Medicine, Department of Occupational Medicine, Dokuz Eylul University, 35330 Izmir, Turkey; 2 Department of Toxicology, Faculty of Pharmacy, Gazi University, 06330 Ankara, Turkey; esraemerce@gazi.edu.tr; 3 Bio Izmir—Izmir Health Technologies Development and Accelerator Research and Application Center, Dokuz Eylul University, 35330 Izmir, Turkey; tungamze@gmail.com; 4 Department of Molecular Medicine, Institute of Health Sciences, Dokuz Eylul University, 35330 Izmir, Turkey; gul.islekel@deu.edu.tr; 5 Vocational School of Health Services, Medical Laboratory Techniques Program, Dokuz Eylul University, 35330 Izmir, Turkey

**Keywords:** healthcare workers, occupational hygiene, genotoxicity, comet, micronucleus test, oxidative stress

## Abstract

Background/Objectives Using genotoxicity tests and oxidative stress indicators, the study evaluated the relationship between healthcare workers’ (HCWs) exposure to chemical risks. This study aimed to evaluate the oxidative damage and genotoxic effects of sub chronic or long-term volatile organic compounds (VOCs) exposure in HCWs. Methods: Pathology workers (Group 1), cleaning workers (Group 2), and medical secretaries (Group 3) were categorized, and VOCs and alkaline dust were measured for Groups 1 and 2 using appropriate occupational hygiene methods. Genotoxicity was assessed using alkaline comet and micronucleus (MN) assays. Oxidative stress indicators were analyzed in first-morning urine samples through liquid chromatography. Results: A total of 90 HCWs participated in the study. The mean R-cdA levels were 0.05 ± 0.02 for medical secretaries, 0.07 ± 0.03 for cleaning workers, and 0.06 ± 0.07 nmol/mmol creatinine for pathology workers (*p* = 0.040). The mean tail intensity (%) was 16.33 ± 10.68 (Group 1), 18.9 ± 7.4 for cleaning workers, and 14.1 ± 6.5 for medical secretaries (*p* = 0.020). Conclusion: Implementing occupational hygiene measures in the working environment has effectively reduced occupational risks. The lack of significant differences in genotoxicity and oxidative stress parameters between the exposed and control groups supports the notion that the exposure limit values are protective.

## 1. Introduction

Over the past decades, healthcare workers [HCWs] have become increasingly concerned about the health hazards posed by chemicals. Disinfectants, sterilizers, anesthesia gases, laboratory reagents, cleaning agents, drugs, and chemotherapeutic agents are the primary sources of chemical risk in the hospital environment. According to the National Institute for Occupational Safety and Health [NIOSH], there are many different chemical hazards that pose risks to healthcare workers [1]. Exposure to these chemical risks can lead to acute or chronic health effects, including respiratory, immunological, carcinogenic, reproductive, cardiovascular, and inflammatory conditions. Acute effects can often be linked to causality more easily due to the short latency period between exposure and the onset of symptoms. However, chronic effects, such as genotoxicity, manifest over many years, with multiple confounding factors making it difficult to establish causal relationships [2].

One of the chronic effects induced by volatile organic compounds [VOCs] in humans is damage to nucleic acids, resulting in oxidative stress, genotoxicity, and inflammation. Reactive oxygen species [ROS] are cytotoxic agents that cause oxidative damage by attacking cell membranes and DNA. Antioxidant mechanisms within the cell can repair some of these genotoxic effects. However, if the damage cannot be repaired, it may lead to cancer [3,4]. For example, Zabadi et al. [5] evaluated the increased incidence of laryngeal, nasopharyngeal, and liver cancers in sewage workers exposed to genotoxic substances such as polycyclic aromatic hydrocarbons [PAHs], benzene and its derivatives, and aldehydes. They found that measuring DNA adducts in peripheral lymphocytes is one of the earliest methods to indicate genotoxicity. Similarly, Chang et al. [6] found high levels of chemical exposure in spray paint workers, which corresponded with increased levels of urine 8-hydroxy-2′-deoxyguanosine [8-OH-dG]. Lamplugh et al. [7] reported a positive correlation between formaldehyde and benzene exposure and genotoxicity tests in nail studio workers, observing significant changes in whole blood comet and buccal epithelial cells micro nucleus [MN] methods.

However, studies on the genotoxicity-oxidative stress in workers occupationally exposed to chemicals have shown contradictory results, with both positive and negative findings reported [3,8,9,10]. Although adverse health effects have been documented, very few studies assess the relationship with subacute or chronic exposures [11,12]. Further studies on DNA damage at different exposure levels are necessary to improve our understanding of the health effects related to occupational exposures. Biomonitoring workers exposed to VOCs using biomarkers of exposure, early genotoxic, oxidative, and inflammatory effects can be valuable tools for early toxicity detection [13]. This approach can also play a crucial role in predicting health risks and preventing the development of diseases.

This study aimed to evaluate the oxidative damage and genotoxic effects of sub chronic or long-term VOCs exposure. To achieve this, we obtained information on healthcare workers’ exposure to chemical risks through occupational hygiene measurements. The relationship between exposure and DNA damage was assessed by evaluating genotoxicity markers using qualitative and quantitative methods.

## 2. Materials and Methods

### 2.1. Population and Sample Selection

The research population comprises 4500 HCWs at the Dokuz Eylul University [DEU] Faculty of Medicine campus. High-risk groups for chemical exposure were identified through Occupational Health and Safety [OHS] Units’ risk assessment results, job analyses, and pre-tests. The OHS Units use 5 × 5 risk matrix methods. A 5 × 5 risk matrix is a type of risk matrix that is visually represented as a table or a grid. It has five categories each for probability [along the X axis] and impact [along the Y axis], all following a scale of low to high. Occupational health professionals categorize workplace risks using a color-coded system based on the likelihood and severity of potential consequences, ranging from green [lowest risk] to purple [highest risk] [Supplemental Tables S1 and S2] [14]. Preliminary measurements were conducted for chemical risks identified as hazardous based on risk assessment and job analysis. The aim was to evaluate these risks both qualitatively and quantitatively, followed by the collection of biological samples from HCWs. The chemical risks measured in the preliminary tests were initially evaluated based on the permissible limit values specified in national legislation [15]. For cases where no national limits were available, the Permissible Exposure Limits [PEL] from the NIOSH Chemical Pocket Guide were used [16]. Based on the pre-test results, pathology department workers [Group 1] and cleaning workers [Group 2] were identified as high-risk groups due to their exposure to VOCs and alkaline dust above permissible exposure limits. Medical secretaries [Group 3] were identified as a low-risk group for VOCs or alkaline dust exposure. For genotoxicity evaluation, the minimum sample size calculated was 26; thus, 30 HCWs per group were recruited, allowing for spare samples.

### 2.2. Sample Selection, Inclusion, and Exclusion Criteria

**Figure 1:**
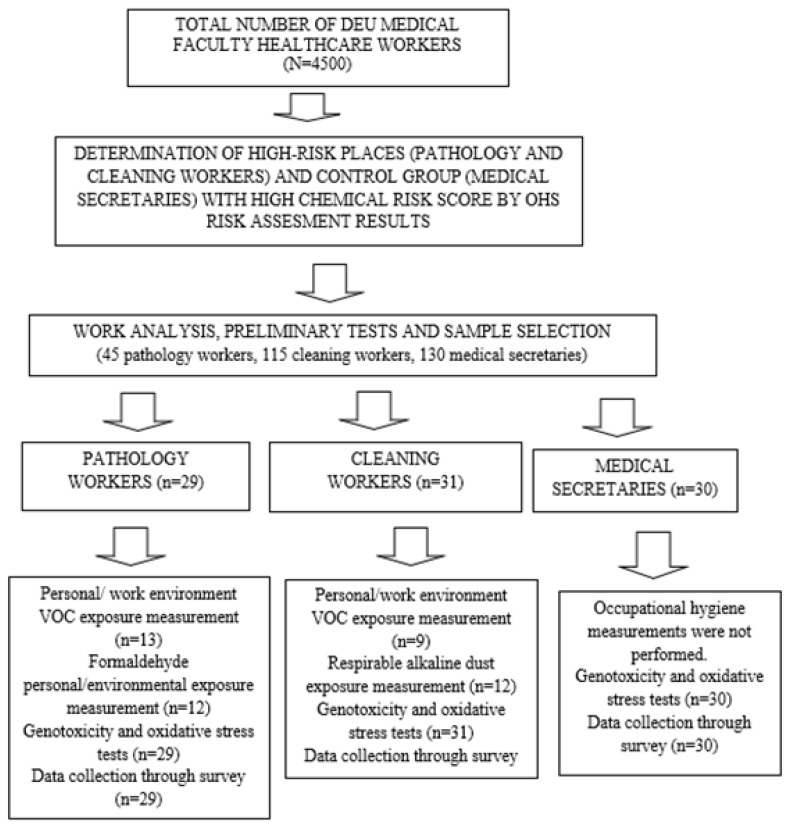
Study Flowchart.

Workers were selected based on their risk level, with a requirement of having worked in the specified department for at least 6 months. Exclusion criteria included individuals who had received cancer treatment at any point, those with a severe infection requiring antibiotics in the last 15 days, and those who were pregnant. Accordingly, 45 workers from Group 1, 115 from Group 2, and 130 from Group 3 met the criteria, and 30 workers from each group were selected voluntarily [Figure 1. Study Flowchart].

### 2.3. Occupational Hygiene Measurements [VOCs and Alkaline Dust Measurement]

The levels of VOCs in the air were measured according to TS ISO 16200-1 [17], while alkaline dust levels were measured following the NIOSH 7401 [18] standard. Before each measurement, a manual device was used to record temperature, humidity, and airflow rate. Additionally, a blank tube was collected from the sampling environment before starting the measurements. For VOCs measurements, personal exposure was assessed using an active sampling pump at a low flow rate of 200 mL/min. Flow verification was performed before each measurement, every 2 h if the measurement period exceeded 2 h, and at the end of the measurement using a rotameter. The samples were then analyzed using a Gas Chromatography-Flame Ionization Detector [GC-FID] (VALMET, ESPOO, FINLAND). For alkaline dust sampling, a 1 µm PTFE membrane filter was used. The pump flow rate was maintained between 1–4 L/min, with a total volume drawn ranging from 70 L to 1000 L. Samples were analyzed using the acid-base titration method as appropriate.

### 2.4. Genotoxicity Tests

Among the genotoxicity tests, MN assay was applied to exfoliated buccal cells, and alkaline comet assay was applied to whole blood. Before collecting biological materials, all workers signed the consent form. Biological samples were collected at the end of the personal exposure measurement day and stored under appropriate conditions.

#### 2.4.1. Buccal MN Method

##### Collection and Analysis of Exfoliated Buccal Cells

Exfoliated buccal epithelial cells were collected from the inside of both cheeks from donors. Two glass slides were prepared for each individual, fixed in 80% methanol for 10 min, and then air-dried at room temperature. The slides were treated with 5M HCl and then Schiff’s reagent [Sigma-Aldrich, Milan, Italy] for 90 min. Air-dried slides were stained with fast green reagent [Sigma-Aldrich, Darmstadt, Germany] and washed with ethanol. The slides were examined at 400× magnification under light microscopy [Primo Star, Zeiss, Germany] for micronuclei. Micronucleated cells were classified according to Bolognesi et al. [19], and their frequencies were reported as number of MN in 1000 differentiated cells.

##### Alkaline Comet Assay

A 1–2 mL peripheral venous blood sample was collected in heparinized tubes from study participants. Obtained whole blood was frozen in aliquotes of 100 µL. On the day of analysis, the blood tubes were quickly thawed at 37 °C. Blood was mixed with 1% low melting agarose and layered on pre-coated slides [with 1% normal melting agarose]. Then the slides were immersed in lysis solution [2.5 M NaCl, 100 mM EDTA, 10 mM Trizma base, 10% DMSO, 1% Triton^®^ X-100] for 2 h. After lysing, the slides were placed in an alkaline electrophoresis buffer [300 mM NaOH, 1 mM Na2EDTA, pH > 13] for 20 min and then electrophoresis was performed at 25 V, 300 mA for another 20 min. Slides were then washed three times with neutralization buffer [0.4 M Tris, pH] and stained with ethidium bromide [20 µg/mL]. Quantitative DNA damage was measured in 200 cells per individual using the “Comet Assay III” image analysis system [Perceptive Instruments, Staffordshire, UK] under a fluorescence microscope [Zeiss Axioscope, Oberkochen, Germany]. DNA damage determination was measured with tail intensity % parameter.

##### Oxidative Stress Tests

The oxidative DNA damage product 8-hydroxy-2-deoxyguanosine [8-OH-dG] appears as a result of oxidation of 2′-deoxyguanosine. Other DNA damage products, [5′R] and [5′S]-8,5′-cyclo-2′-deoxyadenosines [R-cdA and S-cdA], produced by hydroxyl radical attack on 2′-deoxyribose moiety of 2′-deoxyadenosine in DNA followed by 8,5′-intramolecular cyclization and oxidation, indicate concomitant damage to both sugar and base moieties of the same nucleoside in DNA and may play an important part in the development of several diseases [20,21]. DNA repair enzymes excise these damaged products, which are then excreted from the body via urine Assessing DNA damage markers, specifically 8-OH-dG, S-cdA, and R-cdA levels in initial urine samples, remains crucial despite understanding various factors impacting oxidative stress in HCWs [22].

##### Collection and Analysis of Oxidative Stress Indicators

Quantitative analyses of damaged DNA nucleosides from first-morning urine samples were performed using a liquid chromatography triple quadrupole ion trap tandem mass spectrometer [Shimadzu, Kyoto, Japan-4000 QTRAP, AB SCIEX, IL60060, Mundelein, IL, USA] in multiple reaction monitoring [MRM] mode with a turbo V ion spray source. Data analysis was conducted using Analyst Software Version 1.5. Samples were separated using a reversed-phase C18 column [Zorbax SB, CA95051, United StatesAq 2.1 × 150 mm, 3.5 μm] and a guard column [Agilent Eclipse XDB, CA95051, United States, C8 2.1 × 12.5 mm, 5 μm] at a flow rate of 0.3 mL/min. Gradient analysis employed dH2O [A] and acetonitrile [B] containing 0.1% formic acid as mobile phases.

### 2.5. Statistics

Data were analyzed using the SPSS [Statistical Package for Social Sciences] 16.0 software package. Descriptive analyses presented frequency data as number [n] and percentage [%], and numerical data as arithmetic mean ± standard deviation [SD] and median [minimum-maximum]. Categorical data were compared using the Chi-square [χ²] test and Fisher’s exact Chi-square test. The Kolmogorov–Smirnov and Shapiro–Wilk tests assessed the conformity of numerical data to a normal distribution. For non-normally distributed numerical variables, the Mann–Whitney U test was used to compare two groups, and the Kruskal–Wallis test was used for more than two groups. In cases where the Kruskal–Wallis test showed significant differences, post hoc tests with Dunn–Bonferroni correction identified the groups causing the difference. Spearman correlation analysis assessed the relationship between two non-normally distributed numerical variables. Two-way ANOVA evaluated the parameters measured in urine according to demographic characteristics in the worker groups. 

## 3. Results

The mean age of the workers was 40.9 ± 8.7 years, with ages ranging from 22 to 59 years. The majority of the workgroup was female, with 15 male workers [16.7%]. Among all workers, 34.4% were current smokers, while 64.4% had never smoked or had quit. Half of the workers had at least one doctor-diagnosed chronic disease, with asthma [5.6%] and hypertension [5.6%] being the most common, followed by ankylosing spondylitis [2.2%] and anemia [2.2%]. The mean duration of employment was 10.4 ± 8.3 years, ranging from 2 to 38 years. On average, workers worked 41.6 ± 7.8 h per week, with a range from 8 to 60 h. The most commonly used personal protective equipment [PPE] among all workers was latex gloves (Table 1). The results of occupational hygiene measurements conducted in the pathology unit are presented in Table 2. A total of 11 different VOCs and formaldehyde chemicals were detected across all measurements. The average concentration of isopropylbenzene from 13 personal occupational hygiene measurements was 1 ± 1.3 mg/m^3^, while the average concentration of toluene was 2.7 ± 4.1 mg/m^3^. Only three measurements exceeded the PEL. Elevated levels of p-m-xylene were observed in a personal measurement in the histotechnical department and a work environment measurement in the molecular test laboratory. The elevated formaldehyde level was recorded in a work environmental measurement in the macroscopy department. In the macroscopy department, environmental formaldehyde levels were found to exceed the PEL value of 0.05 mg/m^3^. Formaldehyde is classified as a Group 1 carcinogen by the International Agency for Research on Cancer (IARC) and is one of the most well-known carcinogenic substances. Table 2 and Table 3 provide an overview of the chemical classes listed by the IARC [23]. The personal and environmental occupational hygiene measurement results for cleaning workers are shown in Table 3. The average concentration of alkaline dust measured in 12 cleaning workers was 1.4 ± 0.3 mg/m^3^. The average concentration of isopropylbenzene was 0.6 ± 0.2 mg/m^3^, and the average concentration of toluene was 1.2 ± 0.8 mg/m^3^. The comparison results of oxidative DNA damage parameters [nmol/mmol creatinine], tail intensity %, and micronucleus frequency [MN/1000 cells] measured between the study groups are presented in Table 4. The average 8-OH-dG measured in the urine was 2.47 ± 3.08 nmol/mmol creatinine for pathology workers, 2.2 ± 0.8 nmol/mmol creatinine for cleaning workers, and 2.3 ± 0.9 nmol/mmol creatinine for medical secretaries. The mean tail intensity % was 16.33 ± 10.68 for pathology workers, 18.9 ± 7.4 for cleaning workers, and 14.1 ± 6.5 for medical secretaries. The mean frequencies of MN in buccal samples were 2.3 ± 2.2, 1.7 ± 1.3, and 1.5 ± 1.2 for pathology workers, cleaning workers, and medical secretaries, respectively. A significant difference in urine *R*-cdA levels was found between groups [*p* = 0.040], attributed to lower *R*-cdA levels in pathology workers compared to cleaning workers [*p* < 0.05]. A statistically significant difference in tail intensity % was also found between groups [*p* = 0.020] due to higher levels in cleaning workers compared to medical secretaries [*p* = 0.018]. The relationship between genotoxicity and oxidative stress parameters with the main independent variable and other variables for all workers is presented in Table 4. No significant differences were detected in urine oxidative DNA damage levels, tail intensity %, and MN frequency between the exposed and control groups [*p* > 0.05]. However, the mean *S*-cdA levels were 0.04 nmol/mmol creatinine in female workers and 0.02 nmol/mmol creatinine in male workers, showing a significant difference [*p* = 0.001]. A significant difference in urinary *S*-cdA levels was also found among smokers, non-smokers, and quitters [*p* = 0.001]. Urinary *S*-cdA levels were significantly higher in smokers compared to non-smokers [*p* = 0.027] and quitters [*p* = 0.027]. The mean levels of R-cdA in urine were significantly different in those who did not use aprons compared to those who did [*p* = 0.043] [Table 5]. Correlation analyses of MN frequency, tail intensity %, and oxidative DNA damage parameters with sociodemographic data and work-related variables in hospital worker groups did not reveal any statistically significant relationships [Supplementary Tables S3–S7]. Similarly, no statistically significant results were obtained when grouping groups 1 and 2 as the exposure group and group 3 as the control group [Table 6].

## 4. Discussion

Herein, we evaluated the relationship between the exposure of DEU Hospital healthcare workers to chemical risks and genotoxicity and oxidative stress indicators. Cleaning workers and medical pathology laboratory workers, identified as having high exposure in preliminary tests, constituted the exposed group, while medical secretaries served as the control group. Except for the comet test, no differences were observed among the three groups regarding oxidative stress indicators and genotoxic activity assessments. When dividing the participants into the exposed group [cleaning workers and pathology workers, n = 60] and the control group [medical secretaries, n = 30], oxidative stress parameters and genotoxicity markers did not show any differences. The results of our study suggest that there is no difference in the genetic damage between workers exposed to chemical risks below PEL values and those not exposed, supporting the safety of these limit values. The study was conducted during the 2 years following the COVID-19 pandemic, a period when HCWs adherence to occupational hygiene measures was high at both personal and administrative levels [24]. Most workers wore gowns and respiratory protection, and work environments were organized according to national regulations and guidelines, with natural ventilation and maximum PPE usage. These measures led to improved occupational hygiene measurement results, with only three out of 45 measurements [6.6%] exceeding limits, compared to higher pre-test results in cleaning and pathology workers. Therefore, it can be concluded that effective occupational hygiene measures reduce exposure and consequently decrease adverse health effects. Studies evaluating the relationship between exposure to chemical risks and genotoxicity-oxidative stress have produced varying results. Significant changes in genotoxicity were observed in auto painters [11], photocopy workers [25], and furniture makers [26] exposed to chemical risks, as indicated by comet and MN methods. In the plastic industry [4], chemicals such as styrene, benzene, and toluene significantly increased genotoxicity test changes compared to the control group. The common finding in these studies is that chemical exposure levels were above the PEL, suggesting that increased exposure correlates with increased genotoxic effects. This hypothesis aligns with the principle of the biological gradient, which posits that the likelihood of damage increases with the amount of exposure. However, different mechanisms must be considered for studies where occupational hygiene measurements are below the PEL yet still show positive or negative effects on genotoxicity tests. For instance, in the comet method evaluation, tail length was significantly longer in the group exposed to perchloroethylene in the dry-cleaning industry. This study measured simultaneous personal VOCs exposure and found that, even below the allowed value, the levels could still be significantly high, indicating the importance of using the comet method for monitoring workers [27].Cavallo et al. [4] evaluated 17 workers involved in spray painting and found that, despite high chemical risk measurements in the work environment, the metabolites of chemicals were low in the workers’ biological monitoring. They attributed this to the use of PPE by the workers, which provided protection. They also suggested that DNA might be protected due to increased expression of DNA repair proteins in response to low-dose chronic exposures, supporting this claim with a negative correlation between years of study and genotoxicity parameters. Similarly, our study found a strong negative correlation between years of work, weekly working hours, and the frequency of MN [Supplementary Table S3]. Moreover, no correlation was observed between age, smoking quantity, years of work, or weekly working hours and urinary measurements, tail density [%], and micronucleus frequency levels in the exposed group [Supplementary Table S4]. Similarly, no correlation was found between weekly working hours, total years of work, and urinary R-cdA, S-cdA, 8-OH-dG, or tail intensity [%] across all workers [Supplementary Tables S5–S7]. Akhlaghi et al. [28] reported a non-significant difference in the mean number of MN cell counts between exposure and control groups in their study of genotoxicity in 32 pathology laboratory workers and 32 control workers. They noted that previous studies mostly evaluated MN cell frequencies, while their study provided both frequency and number, leading to differences in the interpretation of genotoxicity tests. They also observed a statistically significant increase in MN cells with longer durations of chemical exposure. Our study did not find consistent results in the correlation analysis between genotoxicity and oxidative stress indicators and years of study [see Supplementary Tables]. Akhlaghi et al. [27] based their study on the hypothesis of formaldehyde exposure without conducting occupational hygiene measurements. They suggested that despite the non-significant difference in MN cell counts, workers should still be monitored [29,30]. Oltulu et al. [31] compared two similar groups of 30 HCWs, one group preparing antineoplastic drugs, regarding sociodemographic variables and found no difference in the comet method results. They highlighted that variable such as personal characteristics [e.g., height, weight, smoking habits], genotoxicity assessment methodologies, and the use of PPE like gloves, masks, and goggles are crucial in preventing exposure and influencing genotoxicity outcomes. Aquino et al. [32] found no statistical difference between exposure and control groups in their study, but they observed differences in comet and MN methods’ results between the beginning of the week and the weekend. In pathology workers and control groups evaluated on Monday and Friday, they noted a threefold increase in karyolitic and apoptotic-like cells in the exposed group between these two days. They hypothesized that basal cells died earlier due to irreparable damage, leading to apoptosis pathways. However, since the damage was not yet permanent, no increase in MN was detected. In our study, no special selection could be made regarding the working days due to technical reasons. In our study, biological materials were collected on the same day that occupational hygiene measurements were taken. These findings suggest that genotoxicity tests should be interpreted according to different parameters, such as the number of cells or significant differences between tests performed on different days, indicating a lack of consensus in interpretation. Our study’s cross-sectional design is a limitation, as evaluating mostly long-term exposure effects, such as genotoxicity, in follow-up studies would yield more qualified results. Conducted post-COVID-19 pandemic, the study’s working conditions in the Department of Pathology and cleaning tasks were managed with measures like reduced staff, rotation, and remote work. These measures mean occupational hygiene exposure measurements may not represent pre-pandemic conditions, as the work was carried out at a lower tempo. Additionally, the voluntary selection of participants likely led to a bias, as workers with high awareness of the subject may have been more inclined to participate. A significant limitation of the study is the inability to measure chemical risks individually, as multiple risks coexist simultaneously in the work environment. Since there may be many confounding factors related to people’s nutrition and lifestyle, new studies are needed to evaluate these variables. Consequently, it was not possible to evaluate the specific contribution of each chemical to the causality of genotoxicity. Addressing these limitations requires large-scale studies funded by national authorities. There are still significant gaps in knowledge on many topics regarding chemical risks and genotoxicity and oxidative stress. Various studies yield different results, highlighting the need for standardized methods and interpretations.

## 5. Conclusions

The most effective way to protect workers is through primary protection measures in OHS, which aim to eliminate risks entirely, thereby preventing diseases and accidents. Data from studies focused on secondary protection, such as early diagnosis or screening, should be used to enhance primary prevention efforts. Similar to our study, these secondary protection measures can provide valuable information to support and strengthen primary prevention strategies.

## Figures and Tables

**Table 1:** Comparison of Sociodemographic Characteristics, Habits, and Working Life Characteristics by Worker Groups.

	All Workers (n:90) (%)	Pathology Worker (n = 29) (%)	Cleaning Worker (n = 31) (%)	Medical Secretary (n = 30) (%)	*p*
Age (years) Mean ± SD (min–max) Median	40.9 ± 8.7 (22.0–59.0) 40.0	40.1 ± 8.3 (27.0–59.0) 39.0	41.2 ± 9.6 (22.0–55.0) 43.0	41.3 ± 8.5 (25.0–55.0) 43.0	0.590 *
Gender Female Male	75 (83.3) 15 (16.7)	21 (72.4) 8 (27.6)	27 (87.1) 4 (12.9)	27 (90.0) 3 (10.0)	0.100 **
Smoking Yes No	47 (52.8) 11 (12.4)	9 (31.0) 20 (69.0)	10 (32.3) 21 (67.7)	12 (41.4) 17 (58.6)	0.700 **
Alcohol use Yes No	42 (47.8) 47 (52.2)	18 (62.1) 11 (37.9)	8 (25.8) *** 23 (74.2)	16 (53.3) 14 (46.7)	0.013 **
Chronic disease Yes No	45 (50.0) 44 (48.9)	16 (55.2) 13 (44.8)	17 (54.8) 14 (45.2)	12 (40.0) 18 (60.0)	0.400 **
Doing sports Yes No	17 (18.9) 73 (81.1)	11 (37.9) *** 18 (62.1)	2 (6.5) 29 (93.5)	4 (13.3) 26 (86.7)	0.005 **
Cigarettes (pack*year) Mean ± SD (min–max) Median	8.1 ± 7.4 (0.6–31.0) 5.1	8.6 ± 7.4 (1.0–22.5) 6.5	8.9 ± 9.6 (0.5–30.0) 4.3	6.4 ± 5.3 (0.1–15.0) 7.5	0.727 *
Year of work (year) Mean ± SD (min–max) Median	10.4 ± 8.3 (2.0–38.0) 10.4	11.3 ± 10.6 (2.0–19.5) 10.0	6.1 ± 5.9 (1.0–10.0) 5.0	7.7 ± 7.1 (1.0–25.0) 5.5	0.200 *
Weekly working time (hours) Mean ± SD (min–max) Median	41.6 ± 7.8 (8.0–60.0) 45.0	42.3 ± 8.1 (090–60.0) 45.0	41.7 ± 8.8 (10.0–48.0) 45.0	40.7 ± 6.7 (8.0–45.0) 40.0	0.100 *
Use of latex gloves Yes No	53 (58.9) 36 (40.0)	25 (86.2) 4 (13.8)	27 (87.1) 4 (12.9)	1 (3.3) 29 (96.7) **	<0.001 *
Use of non-latex gloves Yes No	13 (14.4) 77 (86.5)	5 (17.2) 24 (82.8)	8 (25.8) 23 (74.2)	0 (0.0) ** 30 (100.0)	0.014 *
Mask use Yes No	82 (91.1) 8 (8.9)	25 (86.2) 4 (13.8)	28 (90.3) 3 (9.7)	29 (96.7) 1 (3.3)	0.400 *
Apron use Yes No	30 (33.3) 60 (66.7)	19 (65.5) ** 10 (34.5)	10 (32.3) ** 21 (67.7)	1 (3.3) ** 29 (96.7)	<0.001 *

*: Kruskal Wallis Test **: Pearson Chi-square Test ***: Group Where the Difference Originates.

**Table 2:** Department of Pathology Occupational Hygiene Measurement Results (mg/m^3^).

n *	Mean ± SD	(Min–Max)	Median (1st–3rd Quart)	IARC Class of the Chemical
Isopropylbenzene (n = 13)	1.0 ± 1.3	(0.4–5.1)	0.5 (0.5–0.9)	2B
Toluene (n = 13)	2.7 ± 4.2	(0.4–16.4)	1.9 (0.9–2.4)	3
2chlorotoluene (n = 13)	0.9 ± 0.2	(0.6–1.2)	0.8 (0.7–1.0)	3
Trichlorobenzene (n = 13)	0.9 ± 0.1	(0.7–1.3)	0.7 (0.8–1.0)	not listed by IARC
Oxylene (n = 13)	693.4 ± 2417.1	(3.7–8737.2)	16.3 (5.3–24.0)	3
Pmxylene (n = 13)	1858.0 ± 6354.7	(20.7–230.1)	103.4 (47.7–154.1)	3
Ethanol (n = 11)	29.7 ± 3.5	(23.3–34.4)	30.6 (27.6–33.0)	1
Dichlorobenzene (n = 11)	0.9 ± 0.1	(0.7–1.2)	0.9 (0.7–0.9)	2B
Tertbutylbenzene (n = 9)	2.9 ± 0.3	(2.4–3.4)	2.8 (2.6–3.2)	not listed by IARC
Methanol (n = 9)	7.5 ± 4.5	(2.2–15.1)	5.7 (4.8–11.2)	not listed by IARC
Formaldehyde-environmental (n = 8)	0.02 ± 0.01	(0.01–0.05)	0.01 (0.01–0.04)	1
Formaldehyde-personal (n = 4)	0.03 ± 0.01	(0.02–0.05)	0.03 (0.01–0.05)	1
Ethylbenzene (n = 1)	1.6	1.6	1.6	2B
Occupational Hygiene Measurements Personal measurement Work environment measurement	6 7	-		
Number of Measurements on PEL **	3			

*: Number of samples in which the chemical was measured SD: Standard deviation (Standard deviation) ** Reference values for PEL were initially checked in the National Regulation on Health and Safety Measures for Working with Chemical Substances. The NIOSH Pocket Guide to Chemical Hazards standard was used if the relevant parameter was not defined there.

**Table 3:** Occupational Hygiene Measurement Results of Cleaning Workers (mg/m^3^).

n	Mean ± SD	(Min–Max)	Median (1st–3rd Quart)	IARC Class of the Chemical
Alkali Dust (n = 12)	1.4 ± 0.3	(0.8–1.8)	1.3 (1.1–1.6)	not listed by IARC
Isopropylbenzene (n = 9)	0.6 ± 0.2	(0.4–0.8)	0.6 (0.4–0.8)	2B
Toluene (n = 9)	1.2 ± 0.8	(0.2–2.4)	1.2 (0.3–2)	3
Dichlorobenzene (n = 9)	1.1 ± 1.1	(0.4–3.8)	0.7 (0.6–1.3)	2B
Pmxylene (n = 8)	72.9 ± 65.3	(6.6–174.2)	66.5 (7.8–127.1)	3
2chlorotoluene (n = 7)	0.8 ± 0.1	(0.6–0.9)	0.8 (0.7–0.9)	3
Trichlorobenzene (n = 7)	0.8 ± 0.05	(0.7–0.9)	0.8 (0.8–0.8)	not listed by IARC
Oxylene (n = 6)	5.9 ± 3.5	(3.4–12.2)	4.1 (3.6–8.8)	3
Tertbutylbenzene (n = 4)	2.5 ± 0.1	(2.3–2.7)	2.5 (2.4–2.7)	not listed by IARC
Ethanol (n = 4)	27.1 ± 5.4	(24.1–35.1)	24.5 (24.2–32.5)	1
Methanol (n = 3)	2.2 ± 0.4	(1.8–2.6)	2.2 (1.8–)	not listed by IARC
Ethylbenzene (n = 1)	1.1	1.1	1.1	2B
Occupational Hygiene Measurements VOC measurement Alkali dust measurement	9 12			
Number of Measurements on PEL *	-		-	

* Reference values for PEL values were firstly checked in the National Regulation on Health and Safety Measures in Working with Chemical Substances, and if the relevant parameter was not defined, the NIOSH Pocket Guide Chemical Hazards standard was used. SD: Standard deviation (Standard deviation).

**Table 4:** The Relationship Between Sociodemographic and Working Life-Related Variables and Genotoxicity and Oxidative Stress Parameters in The Entire Study Group.

Whole Group (N:90)		8-OH-dG in Urine (nmol/mmol Creatinine) Mean ± SD (min–max) Median	*p*	S-cdA in Urine (nmol/mmol Creatinine) Mean ± SD (min–max) Median	*p*	R-cdA in Urine (nmol/mmol Creatinine) Mean ± SD (min–max) Median	*p*	Tail Intensity% Mean ± SD (min–max) Median	*p*	MN Frequency (MN/1000 Cells) Mean ± SD (min–max) Median	*p*
Exposure status	Exposure group (n = 60)	2.33 ± 2.20 (0.31–17.61) 1.91	0.306 *	0.04 ± 0.03 (0.00–0.21) 0.04	0.849 *	0.06 ± 0.05 (0.00–0.33) 0.05	0.659 *	17.67 ± 9.15 (3.43–44.23) 16.92	0.053 *	2.00 ± 1.82 2.00 (0.00–8.00)	0.279 *
	Control group (n = 30)	2.31 ± 0.93 (0.97–5.03) 2.21		0.04 ± 0.03 (0.00–0.11) 0.04		0.05 ± 0.02 (0.02–0.10) 0.05		14.14 ± 6.52 (5.65–31.62) 11.91		1.46 ± 1.25 (0.00–8.00) 1.00	
Gender	Female (n = 75)	2.38 ± 2.01 (0.31–17.61) 2.19	0.555 *	0.04 ± 0.03 (0.00–0.21) 0.04	0.001 *	0.06 ± 0.04 (0.00–0.33) 0.05	0.772 *	16.30 ± 7.99 (3.43–44.23) 15.38	0.987 *	1.70 ± 1.46 (0.00–7.00) 2.00	0.494 *
	Male (n = 25)	2.05 ± 0.87 (1.17–4.18) 1.91		0.02 ± 0.01 (0.00–0.06) 0.02		0.06 ± 0.04 (0.02–0.16) 0.05		17.45 ± 10.95 (4.21–36.63) 14.83		2.40 ± 2.41 (0.00–8.00) 1.00	
Age group	under 40 (n = 35)	2.65 ± 2.75 (0.88–17.61) 2.16	0.484 *	0.04 ± 0.03 (0.01–0.21) 0.04	0.890 *	0.07 ± 0.06 (0.02–0.33) 0.05	0.564 *	14.55 ± 7.74 (3.57–35.12) 12.97	0.095 *	1.68 ± 1.65 (0.00–8.00) 1.00	0.500 *
	40 and above (n = 55)	2.12 ± 0.93 (0.31–5.32) 1.93		0.04 ± 0.03 (0.00–0.16) 0.04		0.05 ± 0.03 (0.00–0.17) 0.05		17.37 ± 8.78 (3.43–44.23) 16.32		1.90 ± 1.68 (0.00–7.00) 2.00	
Glove use	Yes (n = 53)	2.34 ± 2.32 (0.31–17.61) 1.91	0.233 *	0.04 ± 0.03 (0.00–0.21) 0.04	0.385 *	0.06 ± 0.05 (0.00–0.33) 0.05	0.256 *	17.37 ± 9.38 (3.43–44.23) 16.46	0.281 *	2.01 ± 1.87 (0.00–8.00) 2.00	0.371 *
	No (n = 37)	2.31 ± 0.93 (0.97–5.03) 2.19		0.04 ± 0.03 (0.00–0.11) 0.04		0.06 ± 0.03 (0.02–0.17) 0.05		15.24 ± 6.97 (5.65–31.62) 12.19		1.54 ± 1.30 (0.00–5.00) 1.00	
Mask use	Yes (n = 82)	2.38 ± 1.94 (0.31–17.61) 2.17	0.275 *	0.04 ± 0.03 (0.00–0.21) 0.04	0.726 *	0.06 ± 0.04 (0.00–0.33) 0.05	0.251 *	16.28 ± 8.36 (3.43–44.23) 14.98	0.514 *	1.74 ± 1.61 (0.00–8.00) 1.00	0.186 *
	No (n = 8)	1.80 ± 0.50 (1.19–2.79) 1.73		0.04 ± 0.03 (0.02–0.11) 0.04		0.05 ± 0.04 (0.02–0.16) 0.03		18.70 ± 10.10 (7.13–36.63) 18.24		2.62 ± 2.06 (0.00–6.00) 2.50	
Apron use	Yes (n = 30)	2.03 ± 0.97 (0.31–5.32) 1.84	0.290 *	0.04 ± 0.02 (0.01–0.12) 0.04	0.546 *	0.05 ± 0.04 (0.00–0.27) 0.05	0.043 *	14.87 ± 9.72 (3.43–35.12) 12.78	0.108 *	1.75 ± 1.80 (0.00–8.00) 1.00	0.616 *
	No (n = 60)	2.47 ± 2.17 (0.94–17.61) 2.20		0.04 ± 0.03 (0.00–0.21) 0.04		0.06 ± 0.04 (0.02–0.33) 0.05		17.31 ± 7.77 (5.62–44.23) 15.85		1.85 ± 1.61 (0.00–7.00) 2.00	
Presence of chronic disease	Yes (n = 32)	2.16 ± 0.98 (0.31–5.32) 2.02	0.326 *	0.04 ± 0.03 (0.00–0.16) 0.04	0.288 *	0.06 ± 0.05 (0.00–0.27) 0.05	0.621 *	17.19 ± 9.27 (4.21–44.23) 16.46	0.617 *	1.86 ± 1.67 (0.00–8.00) 2.00	0.669 *
	No (n = 58)	2.49 ± 2.45 (0.94–17.61) 1.92		0.04 ± 0.03 (0.00–0.21) 0.04		0.06 ± 0.04 (0.02–0.33) 0.05		15.86 ± 7.75 (3.43–36.63) 14.98		1.77 ± 1.69 (0.00–7.00) 1.00	
Continuous drug use	Yes (n = 42)	2.05 ± 0.95 (0.31–5.32) 1.86	0.872 *	0.04 ± 0.03 (0.00–0.16) 0.03	0.386 *	0.06 ± 0.05 (0.00–0.27) 0.05	0.958 *	17.18 ± 9.13 (4.53–44.23) 15.88	0.673 *	1.71 ± 1.41 (0.00–4.00) 2.00	0.993 *
	No (n = 48)	2.48 ± 2.21 (0.88–17.61) 2.24		0.04 ± 0.03 (0.00–0.21) 0.04		0.06 ± 0.04 (0.02–0.33) 0.05		16.12 ± 8.17 (3.43–36.63) 14.98		1.87 ± 1.80 (0.00–8.00) 1.00	
Doing sports	Yes (n = 17)	2.04 ± 0.75 (1.17–3.86) 1.86	0.610 *	0.03 ± 0.02 (0.02–0.11) 0.03	0.321 *	0.07 ± 0.06 (0.02–0.27) 0.05	0.241 *	14.69 ± 7.71 (5.65–35.12) 12.60	0.330 *	2.29 ± 2.49 (0.00–8.00) 1.00	0.785 *
	No (n = 73)	2.39 ± 2.04 (0.31–17.61) 2.02		0.04 ± 0.03 (0.00–0.21) 0.04		0.06 ± 0.04 (0.00–0.33) 0.05		16.92 ± 8.66 (3.43–44.23) 15.44		1.70 ± 1.40 (0.00–6.00) 2.00	
Alcohol use	Yes	2.61 ± 2.54 (1.01–17.61) 2.24	0.302 *	0.04 ± 0.03 (0.00–0.21) 0.04	0.568 *	0.06 ± 0.06 (0.02–0.33) 0.05	0.589 *	17.26 ± 9.02 (3.43–44.23) 15.29	0.518 *	2.02 ± 2.05 (0.00–8.00) 1.00	0.833 *
	No	2.07 ± 0.91 (0.31–4.18) 1.89		0.04 ± 0.02 (0.00–0.16) 0.04		0.06 ± 0.03 (0.00–0.17) 0.05		15.83 ± 8.03 (3.57–34.60) 15.04		1.63 ± 1.22 (0.00–5.00) 2.00	
Smoking	Yes (n = 31)	2.68 ± 2.88 (1.19–17.61) 2.24	0.627 **	0.06 ± 0.04 (0.01–0.21) 0.05	0.006 **	0.06 ± 0.05 (0.02–0.33) 0.05	0.996 **	16.45 ± 7.51 (3.43–36.63) 15.38	0.481 **	2.30 ± 2.05 (0.00–8.00) 2.00	0.155 **
	No (n = 48)	2.10 ± 0.91 (0.88–5.03) 1.91		0.03 ± 0.02 (0.00–0.16) 0.04		0.06 ± 0.02 (0.00–0.16) 0.04		15.68 ± 7.95 (3.57–31.62) 14.83		1.46 ± 1.34 (0.00–5.00) 1.00	
	Quit (n = 11)	2.36 ± 1.26 (0.31–5.32) 2.37		0.03 ± 0.03 (0.00–0.11) 0.02		0.06 ± 0.04 (0.00–0.16) 0.05		20.80 ± 12.35 (4.53–44.23) 20.21		2.09 ± 1.57 (0.00–4.00) 2.00	
Working Years	<5 years (n =34)	2.62 ± 2.78 (0.88–17.61) 2.19	0.647 *	0.05 ± 0.04 (0.00–0.21) 0.04	0.074 *	0.06 ± 0.05 (0.02–0.33) 0.05	0.431 *	17.17 ± 7.49 (3.57–31.62) 16.36	0.314 *	1.94 ± 1.63 (0.00–7.00) 1.50	0.509 *
	>5 years (n = 56)	2.15 ± 0.96 (0.31–5.32) 1.92		0.04 ± 0.02 (0.00–0.16) 0.04		0.06 ± 0.04 (0.00–0.27) 0.05		16.08 ± 9.09 (3.43–44.23) 12.83		1.74 ± 1.70 (0.00–8.00) 2.00	

SD: Standard deviation (Standard deviation) *: Mann Whitney U Test **: Kruskal Wallis Test.

**Table 5:** Comparison of Urinary Oxidative Stress Parameters and Genotoxicity Test Results of Study Groups.

	Pathology Worker (n = 29) Mean ± SD (Min–Max) Median	Cleaning Worker (n = 31) Mean ± SD (Min–Max) Median	Medical Secretary (n = 30) Mean ± SD (Min–Max) Median
8-OH-dG in urine (nmol/mmol creatinine)	2.4 ± 3.1 (0.3–17.6) 1.7	2.2 ± 0.8 (0.9–4.0) 2.0	2.3 ± 0.9 (0.9–5.0) 2.2
S-cdA in urine (nmol/mmol creatinine)	0.04 ± 0.03 (0–0.2) 0.03	0.04 ± 0.03 (0.01–0.2) 0.04	0.04 ± 0.03 (0.0–0.1) 0.04
R-cdA in urine (nmol/mmol creatinine)	0.06 ± 0.07 (0–0.3) ** 0.05	0.07 ± 0.03 (0.02–0.17) 0.06	0.05 ± 0.02 (0.02–0.1) 0.05
Tail intensity %	16.3 ± 10.7 (3.4–44.2) 14.8	18.9 ± 7.4 (3.6–0.1) ** 20.2	14.1 ± 6.5 (5.6–31.6) 11.9
MN Frequency (MN/1000 cells)	2.3 ± 2.2 (0.0–8.0) 2.0	1.7 ± 1.3 (0.0–5.0) 2.0	1.5 ± 1.2 (0.0–5.0) 1.0

** Group where the difference originates.

**Table 6:** Comparison of Urine Measurements, Tail intensity %, and MN Frequency Levels Between Exposed and Control Groups.

	Exposure Group (n = 60)	Control Group (n = 30)	*p* *
8-OH-dG in urine (nmol/mmol) Mean ± SD (min–max) Median	2.33 ± 2.20 (0.31–17.61) 1.91	2.31 ± 0.93 (0.97–5.03) 2.21	0.306
S-cdA in urine (nmol/mmol) Mean ± SD (min–max) Median	0.04 ± 0.03 (0.00–0.21) 0.04	0.04 ± 0.03 (0.00–0.11) 0.04	0.849
R-cdA in urine (nmol/mmol) Mean ± SD (min–max) Median	0.06 ± 0.05 (0.00–0.33) 0.05	0.05 ± 0.02 (0.02–0.10) 0.05	0.659
Tail intensity %	17.67 ± 9.15 (3.43–44.23) 16.92	14.14 ± 6.52 (5.65–31.62) 11.91	0.053

*: Mann Whitney U Test.

## Data Availability

Data set can be obtained from the responsible author upon request.

## Supplementary Materials

The following supporting information can be downloaded at: https://www.mdpi.com/article/10.3390/toxics13030189/s1, Table S1: Pre-test results of occupational health and safety unit’s risk assessment scores (colored) and Volatile Organic Compounds (VOCs) and alkaline dust measurement (numbered); Table S2: 5*5 risk assessment of occupational health and safety unit at Dokuz Eylul University Hospital; Table S3: The Relationship of Tail intensity % Levels with Sociodemographic and Urinary Parameters and Micronucleus Frequency; Table S4: The Relationship of Age, Amount of Smoking, Years of Employment, and Weekly Working Hours with Urinary Measurements, Tail Intensity %, and Micronucleus Frequency Levels in the Exposure Group; Table S5: Relationship between 8-OH-dG levels measured in urine and sociodemographic and other urinary parameters. Table S6: The relationship between *R*-cdA levels measured in urine and sociodemographic characteristics; Table S7: The relationship between *S*-cdA levels measured in urine and sociodemographic and other urinary parameters

## Abbreviations

VOC	Volatile Organic Compounds
ROS	Reactive oxygen species
MN	Micronucleus
NIOSH	National Institute for Occupational Safety and Health
PAH	Polycyclic aromatic hydrocarbons
IARC	International Agency for Research on Cancer
