# Supplementary material for: Chemical Risks, Genotoxicity, and Oxidative Stress in Healthcare Workers
Source: Toxics. 2025 Mar 6;13(3):189. doi: 10.3390/toxics13030189 (PMC11946744; doi:10.3390/toxics13030189)
Supplement: Supplementary file 1 [file toxics-13-00189-s001.zip › toxics-3436285-supplementary.pdf]

Supplementary Table S1. Pre-test results of occupational health and safety unit' risk assessment scores (colored) and Volatile Organic Compounds (VOC s) and alkaline dust measurement (numbered)

Place	Unit	RD result score	Substance detected as a result of measurement	Time weighted average value **	Permissible limit value
Central Laboratory	Genetics Laboratory	Methanol 15.7<br>Xylene 15.7	Toluene<br>p.m.-xylene	0.09<br>0.12	192***<br>221***
Operating room	Compilation	6.9			
	Halls	7.4	1.2.4 trichlorobenzene<br>Toluene	0.21<br>0.06	15.1***<br>192***
	PACU*	-	Toluene<br>Ethanol	0.08<br>Trace amount	192***<br>1900****
	Cystoscopy	-	Toluene	0.12	192***
Cleaning Workers	Alkaline dust	-	42.89	2.00	
	Total VOC	11.2	Benzene<br>Toluene<br>Tertbutyl benzene<br>n-Hexane	0.026<br>0.031<br>0.020<br>0.069	3.25*****<br>192***<br>-<br>72***
Medical Pathology Laboratory	Macroscopy	16.8	Formaldehyde	0.400	0.02****
	Histochemistry	16.8	Benzene<br>Toluene<br>Tetrachloroethane<br>Ethylbenzene<br>p.m.-xylene<br>Styrene<br>n-Hexane	0.029<br>0.062<br>0.211<br>0.290<br>1.026<br>0.025<br>0.013	3.25****<br>192***<br>678****<br>442***<br>221***<br>215****<br>72***
Central sterilization	Dirty area<br>Washing hall	11.4	Tert butyl benzene Isopropyl alcohol (2-propanol)<br>n-Hexane	0.00086077<br>0.000443063<br>0.000706787	-<br>-<br>72***

*PACU: Post-anesthesia intensive care unit

**Time-weighted average value is calculated over 8 hours.

*** According to the National Regulation on Health and Safety Precautions in Working with Chemical Substances

**** According to NIOSH Pocket Guide Chemical Hazards standard

****Calculated according to the Regulation on Health and Safety Measures at Work with Carcinogenic or Mutagenic Substances.

Supplementary Table S2. 5*5 risk assessment of occupational health and safety unit at Dokuz Eylul University Hospital

COLOR	POINT	DESCRIPTION
GREEN	0.1 - 1.5	The factor is in the environment but does not generate risks.
BLUE	1.6 - 6.5	The risk factor is present, and it is under control or does not result in unrepairable persistent consequences.
YELLOW	6.6 - 12.5	The factor could lead to health problems or outcomes. It might generate problems during control.
RED	12.6 – 20.5	The factor could cause open and urgent health problems. Precaution is absolutely important.
PURPLE	20.6 – 25.0	The control of the fact matters vital, consequences could be lethal. It is not possible to work on that risk level.

COLOR POINT DESCRIPTION

GREEN 0.1 - 1.5 The factor is in the environment but does not generate risks.

BLUE 1.6 - 6.5 The Risk factor is present, and it is under control or does not result in unrepairable persistent consequences.

YELLOW 6.6 - 12.5 The factor could lead to health problems or outcomes. It might generate problems during control.

RED 12.6 – 20.5 The factor could cause open and urgent health problems. Precaution is absolutely important.

PURPLE 20.6 – 25.0 The control of the fact matters is vital, and the consequences could be lethal. It is not possible to work on that risk level.

Supplementary Table S3. The Relationship of Tail intensity % Levels with Sociodemographic and Urinary Parameters and Micronucleus Frequency

Tail Intensity (%)	Pathology worker		Cleaning worker		Medical Secretary	
	r	p*	r	p*	r	p*
Age (yrs)	0,036	0,853	0,464	0,009	0,124	0,515
Smoking (pack*yr)	-0,341	0,232	-0,154	0,599	-0,350	0,322
Years of employment	0,177	0,360	0,185	0,320	0,123	0,534
Weekly working hours (hrs)	-0,124	0,539	0,053	0,778	-0,009	0,965
Urine 8-OH-dG (nmol/mmol creatinin)	-0,220	0,252	0,227	0,220	0,088	0,644
Urine *S*-cdA (nmol/mmol creatinin)	-0,459	0,012	-0,097	0,605	-0,253	0,178
Urine *R*-cdA (nmol/mmol creatinin)	-0,183	0,341	-0,098	0,599	-0,146	0,440
Micronucleus Frequency (MN cells/1000 cells)	0,441	0,017	0,256	0,172	0,091	0,634

*: Spearman Correlation Test

Supplementary Table S4. The Relationship of Age, Amount of Smoking, Years of Employment, and Weekly Working Hours with Urinary Measurements, Tail Intensity %, and Micronucleus Frequency Levels in the Exposure Group

Exposure Group(n=60)		Age (yrs)	Smoking (pack*yr)	Years of employment	Weekly working (hrs)
Urine 8-OH-dG (nmol/mmol creatinin)	r	0,103	0,080	0,040	0,047
	p	0,434	0,680	0,760	0,727
Urine *S*-cdA (nmol/mmol creatinin)	r	-0,012	0,364	0,155	0,008
	p	0,930	0,052	0,236	0,954
Urine *R*-cdA (nmol/mmol creatinin)	r	0,005	0,055	0,076	-0,139
	p	0,972	0,776	0,561	0,296
Tail DNA LengthKuyruk (%)	r	0,302	-0,224	0,194	0,018
	p	0,019	0,244	0,137	0,891
Micronucleus Frequency(MN cells/1000 cells)	r	0,181	-0,164	-0,006	-0,242
	p	0,169	0,405	0,967	0,070

*: Mann Whitney-U Test

Supplementary Table S5. Relationship between 8-OH-dG levels measured in urine and sociodemographic and other urinary parameters

8-OH-dG in urine	Pathology Worker		Cleaning Worker		Medical Secretary	
	r	p*	r	p*	r	p*
Age (years)	0.008	0.968	0.163	0.381	-0.133	0.484
Cigarettes (pack*year)	0.421	0.134	0.170	0.561	-0.436	0.208
Working Years	-0.099	0.608	0.135	0.469	0.04	0.983
Weekly Working Time (hours)	0.176	0.379	-0.146	0.433	-0.239	0.211
S-cdA in urine (nmol/mmol creatinine)	0.165	0.392	0.068	0.715	0.367	0.046
R-cdA in urine (nmol/mmol creatinine)	0.539	0.003	0.401	0.025	0.235	0.212

*: Spearman Correlation Test

Supplementary Table S6. The relationship between *R*-cdA levels measured in urine and sociodemographic characteristics

R-cdA in urine	Pathology Worker		Cleaning Worker		Medical Secretary	
	r	p*	r	p*	r	p*
Age (years)	0.075	0.700	-0.074	0.691	-0.027	0.889
Cigarettes (pack*year)	0.324	0.259	0.429	0.126	-0.013	0.973
Working Years	-0.077	0.690	0.059	0.751	-0.188	0.338
Weekly Working Time (hours)	-0.227	0.255	-0.160	0.391	-0.312	0.099

*: Spearman Correlation Test

Supplementary Table S7. The relationship between *S*-cdA levels measured in urine and sociodemographic and other urinary parameters

S-cdA in urine	Pathology Worker		Cleaning Worker		Medical Secretary	
	r	p*	r	p*	r	p*
Age (years)	-0.040	0.837	-0.027	0.884	-0.085	0.654
Cigarettes (pack*year)	0.355	0.213	0.431	0.124	0.446	0.197
Working Years	-0.004	0.983	-0.222	0.230	-0.052	0.794
Weekly Working Time (hours)	-0.349	0.075	0.243	0.187	-0.062	0.748
R-cdA in urine (nmol/mmol creatinine)	0.138	0.476	0.335	0.065	0.380	0.038

*: Spearman Correlation Test
